# Supplementary material for: Nanomechanical action opens endo-lysosomal compartments
Source: Nat Commun. 2023 Oct 20;14:6645. doi: 10.1038/s41467-023-42280-9 (PMC10589329; doi:10.1038/s41467-023-42280-9)
Supplement: Supplementary file 1 — Supplementary Information [file 41467_2023_42280_MOESM1_ESM.pdf]

Supplementary Information

Nanomechanical Action Opens Endo−Lysosomal Compartments

Yu Zhao, Zhongfeng Ye, Donghui Song, Douglas Wich, Shuliang Gao, Jennifer Khirallah, Qiaobing Xu*

Department of Biomedical Engineering, Tufts University
Medford, MA 02155 (USA)
Correspondence should be addressed to Q.X. (email: qiaobing.xu@tufts.edu).

Table of Content

1. Instrument 1
2. Synthesis of SAzo and TAzo lipidoids 1
2.1 Synthesis of Compound 1 1
2.2 Synthesis of Compound 2 2
2.3 Synthesis of Compound 3 2
2.4 Synthesis of Compound 4 2
2.5 Synthesis of SAzo lipidoid[1] 3
2.6 Synthesis of TAzo lipidoid 3
3. The photoisomerization of SAzo and TAzo lipidoids 4
4. Characterization of SAzo-LNMs and TAzo-LNMs 4
4.1 TEM measurements 4
4.2 DLS measurements 4
5. Quantification of cytosolic transport efficiency 4
6. TAzo-LNMs facilitate cross-presentation of tumour antigens 5
7. Inhabitation of tumour metastasis to the lung 5
8. Statistical Analysis 6
9. Supporting results 7
10. References 22

Supplementary Figure 1 | Synthesis of SAzo and TAzo lipidoid.
Supplementary Figure 2 | Schematic diagram of the SLEEQ assay.
Supplementary Figure 3 | Characterization of Compound 2 and Compound 3.
Supplementary Figure 4 | Characterization of SAzo lipidoid.
Supplementary Figure 5 | Characterization of TAzo lipidoid.
Supplementary Figure 6 | The photoisomerization of Azo-based lipidoids and their LNM formulations.
Supplementary Figure 7 | Live-cell imaging experiments show the transport capability of LNMs (TAzo-LNMs).
Supplementary Figure 8 | Co-localization analysis of the transport capability of SAzo-LNMs and TAzo-LNMs.
Supplementary Figure 9 | Characterization of GFP mRNA-loaded SAzo-LNMs and TAzo-LNMs.
Supplementary Figure 10 | LNMs transport GFP mRNA from endo−lysosomal compartments to cytoplasm.
Supplementary Figure 11 | LNMs destabilize the endo−lysosomal membrane and induce membrane disruption.
Supplementary Figure 12 | LNMs transport Cre proteins from endo−lysosomal compartments to cytoplasm.
Supplementary Figure 13 | Analysis of DC maturation.
Supplementary Figure 14 | Antitumour effect in a mouse model of melanoma.
Supplementary Figure 15 | Gating strategy for identification of lymphocytes.
Supplementary Figure 16 | Antitumour immunity *in vivo*.
Supplementary Figure 17 | Western blots assay.

1. Instrument

All ^{1}H nuclear magnetic resonance (^{1}H NMR) spectra were recorded on a Bruker AVIII 500 MHz NMR spectrometer operated in the Fourier transform mode. ESI-MS spectra was collected by Finningan LTQ. Hydrodynamic size (Dynamic light scattering measurements, DLS) and polydispersity index of nanoparticles were measured by a Zeta-PALS particle size analyzer (Brookhaven Instruments). Transmission Electron Microscopy (TEM) measurements were performed on a FEI Technai Spirit Transmission Electron Microscope. The cells were observed using confocal laser scanning microscopy (Eclipse Ti2, Nikon, Japan). Flow cytometry analysis was performed on a BD Accuri C6 flow cytometry (BD, USA). *In vivo* imaging of the mice was performed on PerkinElmer® IVIS® Systems (Bioluminescence Imaging).

2. Synthesis of SAzo and TAzo lipidoids

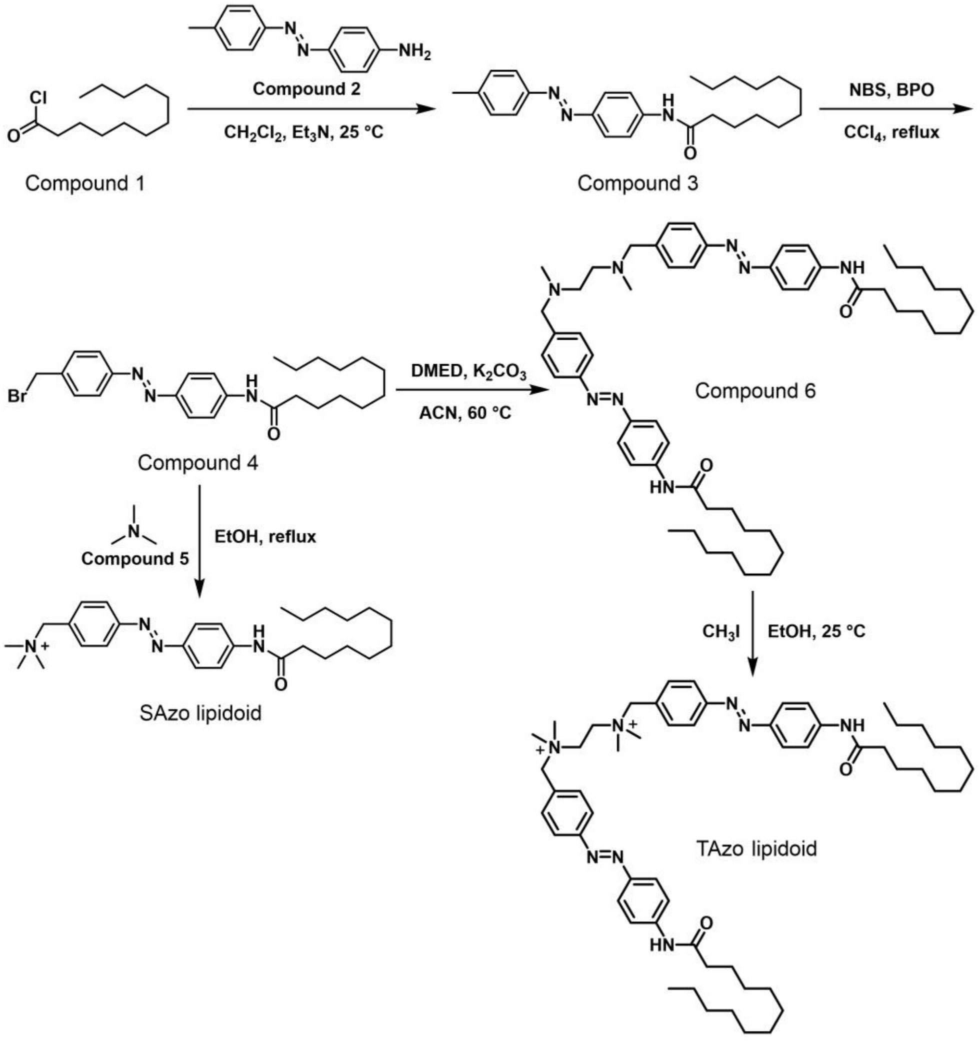

Supplementary Figure 1 | Synthesis of SAzo and TAzo lipidoid.

2.1 Synthesis of Compound 1

Dodecanoic acid (2.0 g, 10 mmol) was suspended in thionyl chloride ($SOCl_2$, 8 mL), then the mixture was stirred at 65 °C overnight (16 hours). After evaporating the excess thionyl chloride, the crude **Compound 1** was obtained as a colorless oil (1.7 g, 7.82 mmol, 7.82% yield).

2.2 Synthesis of Compound 2

To a stirring solution of *p*-Toluidine (1.1 g, 10 mmol, 1.0 eq) in 20 mL of dichloromethane (CH_2Cl_2) was added a solution of Oxone® (12.3 g, 20 mmol, 2.0 eq) in water (30 mL). The reaction mixture was then stirred for 6 hours at room temperature in the absence of light. Subsequently, the deep green coloured organic layer was separated, the aqueous solution was neutralized by the addition of saturated solution of sodium bicarbonate ($NaHCO_3$, 50 mL) and extracted with CH_2Cl_2 (3 × 30 mL). The combined organic layers were dried with anhydrous magnesium sulfate ($MgSO_4$), filtered off and the solvent was removed in reduced pressure. The crude nitroso-compound was then dissolved in a mixture of dry CH_2Cl_2 (20 mL). To this solution *p*-phenylenediamine (1.1 g, 10 mmol, 1.0 eq) and glacial acetic acid (15 mL) was added. The mixture was finally stirred overnight (16 hours) in the absence of light, the solvent was then evaporated under reduced pressure and the residue was purified by column chromatography (hexane/ethyl acetate, 3/1 to 1/1, v/v) to yield **Compound 2** (1.4 g, 6.8 mmol, 67.9% yield) as an orange solid. TLC: R*f* = 0.59 (hexane/ethyl acetate, 3/1, v/v).

^{1}H NMR ($CDCl_3$, 500 MHz, ppm): δ 7.82 (d, J=10.0 Hz, 2H), 7.78 (d, J=10.0 Hz, 2H), 7.30 (d, J=10.0 Hz, 2H), 6.77 (d, J=10.0 Hz, 2H), 4.04 (s, 2H), 2.44 (s, 3H). ESI-MS: m/z calculated for [M + H^+] $C_{13}H_{13}N_3$: 211.11; found: 212.23.

2.3 Synthesis of Compound 3

Compound 2 (1.5 g, 5.8 mmol, 1.0 eq) was dissolved in dry CH_2Cl_2 (30 mL) and triethylamine (Et_3N, 1.2 g, 11.6 mmol, 2.0 eq) was added to the solution. Compound 1 (1.9 g, 8.8 mmol, 1.5 eq) was dissolved in dry CH_2Cl_2 (10 mL) and then it was added dropwise to the above solution of compound 2. After stirring overnight (16 hours) at 25 °C, the solvent was removed under reduced pressure to yield a brown solid. The residue was purified by column chromatography (hexane/ethyl acetate, 5/1 to 1/1, v/v) to yield **Compound 3** (1.9 g, 4.8 mmol, 82.9% yield) as an orange solid. TLC: R*f* = 0.45 (hexane/ethyl acetate, 5/1, v/v).

^{1}H NMR ($CDCl_3$, 500 MHz, ppm): δ 7.92 (d, J=10.0 Hz, 2H), 7.83 (d, J=10.0 Hz, 2H), 7.71 (d, J=10.0 Hz, 2H), 7.38 (s, 1H), 7.32 (d, J=10.0 Hz, 2H), 2.45 (s, 3H), 2.43–2.36 (m, 2H), 1.81–1.75 (m, 2H), 1.42–1.28 (m, 16H), 0.91 (t, J=5.0 Hz, 3H). ESI-MS: m/z calculated for [M + H^+] $C_{25}H_{35}N_3O$: 394.28; found: 394.38.

2.4 Synthesis of Compound 4

Compound 3 (0.5 g, 1.3 mmol, 1.0 eq), N-Bromosuccinimide (NBS, 0.35 g,1.9 mmol, 1.5 eq), and benzoyl peroxide (BPO, 15 mg) were dissolved in carbon tetrachloride (CCl_4, 15 mL) under an argon atmosphere, then the reaction mixture was heated at reflux for 48 hours. The Compound 4 and Compound 3 have the same R*f*

value based on TLC, which was confirmed by the ^{1}H NMR spectrum. When ^{1}H NMR spectroscopy showed that the reaction was almost completed, the mixture was cooled to 0 °C and filtered under reduced pressure to yield a yellow solid, which was washed with cold diethyl ether (3 × 20 mL). Finally, the unpurified **Compound 4** was obtained as a yellow solid (0.3 g).

2.5 Synthesis of SAzo lipidoid[1]

A solution of unpurified Compound 4 (0.5 g, 1.1 mmol, 1.0 eq) in ethanol (10 mL) and trimethylamine (Me_3N, Compound 5, 45% in water, 3.0 mL, >10 eq) was stirred at reflux temperature for 24 hours under an argon atmosphere. The solution was concentrated under reduced pressure. The residue was diluted with ice diethyl ether/CH_2Cl_2 (5/1, v/v, 15.0 mL), the orange precipitate was collected by filtration and washed with ice diethyl ether/CH_2Cl_2 (5/1, v/v) to remove unreacted Compound 4 and Compound 5, and then the desired **SAzo lipidoid** was obtained as an orange solid (0.3 g, 0.8 mmol, 66.3% yield).

^{1}H NMR (CD_3OD, 500 MHz, ppm): δ 8.05 (d, J=5.0 Hz, 2H), 7.96 (d, J=10.0 Hz, 2H), 7.83 (d, J=5.0 Hz, 2H), 7.77 (d, J=5.0 Hz, 2H), 4.64 (s, 2H), 3.19 (s, 9H), 2.44 (t, J=5.0 Hz, 2H), 1.76–1.73 (m, 2H), 1.40–1.30 (m, 16H), 0.91 (t, J=5.0 Hz, 3H). ^{13}C NMR (CD_3OD, ppm): δ 173.8, 153.9, 148.6, 142.4, 133.8, 129.7, 123.7, 122.8, 119.6, 68.6, 52.0, 36.7, 31.7, 29.3, 29.2, 29.1, 28.9, 25.4, 22.3, 13.1. ESI-MS: m/z calculated for [M^+] $C_{28}H_{43}N_4O$: 451.34; found: 451.70.

2.6 Synthesis of TAzo lipidoid

TAzo lipidoid was synthesized via an one-pot method. Unpurified Compound 4 (1.0 g, 2.1 mmol, 1.0 eq), potassium carbonate (K_2CO_3) and N,N'-dimethylethylenediamine (DMED, 18.5 mg, 0.2 mmol, 0.1 eq) were dissolved in anhydrous acetonitrile (40 mL) and stirred at 60 °C for 30 hours under an argon atmosphere. TLC: R*f* = 0.60 (hexane/ethyl acetate, 5/1, v/v). The **Compound 6** was confirmed by HPLC-ESI-MS (**Fig. S3a**). The solution was concentrated under reduced pressure and dissolved in 40 mL of ethanol. Iodomethane (0.6 g, 4.2 mmol, 2 eq) was added into the above solution and stirred at 25 °C for 24 hours under an argon atmosphere. The solution was then concentrated under reduced pressure. The residue was diluted with ice diethyl ether/CH_2Cl_2 (5/1, v/v, 30.0 mL), the red-brown precipitate was collected by filtration and washed with ice diethyl ether/CH_2Cl_2 (5/1, v/v, 3 × 20 mL). The desired **TAzo lipidoid** was obtained as a red-brown colored solid (0.10 g, 0.11 mmol, 10.5% yield).

^{1}H NMR (CD_3OD, 500 MHz, ppm): δ 8.04 (d, J=5.0 Hz, 4H), 7.96 (d, J=10.0 Hz, 4H), 7.82 (d, J=5.0 Hz, 4H), 7.76 (d, J=5.0 Hz, 4H), 4.73 (s, 2H), 3.97 (s, 4H), 3.18 (s, 12H), 2.45 (t, J=5.0 Hz, 4H), 1.74 (m, 4H), 1.40–1.31 (m, 32H), 0.91 (t, J=5.0 Hz, 6H). ^{13}C NMR (CD_3OD, ppm): δ 173.7, 154.0, 148.6, 142.4, 133.7, 129.7, 123.7, 122.8, 119.7, 69.2, 64.4, 51.9, 36.7, 31.6, 29.3, 29.2, 29.0, 28.9, 25.4, 22.3, 13.0. ESI-MS: m/z calculated for [$M/2^+$] $C_{56}H_{84}N_8O_2$: 450.34; found: 450.45.

3. The photoisomerization of SAzo and TAzo lipidoids

SAzo lipidoid was prepared as 30 μM solution in anhydrous dimethyl sulfoxide (DMSO). TAzo lipidoid was prepared as 15 μM solution in DMSO. These samples were irradiated with UV (365 nm for 6 s) and Vis light (>400 nm for 6 s), respectively. The power densities for 365 and >400 nm are 5.40 and 20 mW/cm^2, respectively.

4. Characterization of SAzo-LNMs and TAzo-LNMs

4.1 TEM measurements

To prepare the TEM sample, 10 μL sample solution was dropped onto a carbon-coated copper grid for 10~15 min and blotted with filter paper to remove excess liquid. Then the sample was negatively stained with phosphotungstic acid (EPTA) (5~10 μL) for 1~2 min, blotted again and air-dried before analysis on TEM.

4.2 DLS measurements

The hydrodynamic size of different formulations was evaluated in PBS at 37 °C.

5. Quantification of cytosolic transport efficiency

HeLa cells (4×10^5 cells per well) were seeded in a 6-well plate and incubated overnight for cell attachment. The next day, cells were transfected with 0.5 μg of the LgBiT expression vector. After overnight incubation, cells were reseeded in a white 96-well plate and incubated overnight for cell attachment.

The SLEEQ assay was then performed according to the following procedure[2].

(i) HiBiT-loaded different formulations were added to the cells.

(ii) After incubation for 4 hours, the cells received UV/Vis light irradiation for 10 min. Excess HiBiT was removed, followed by another 30 min incubation. Addition of substrate allowed the measurement of the luminescence from complemented HiBiT/LgBiT in cytoplasm.

(iii) To take into account any luminescent signal from excreted LgBiT, the cell culture medium was collected and measured.

(iv) To determine the total association, digitonin was added to the HiBiT-treated cells to permeabilise cell membranes. This enabled any HiBiT trapped in endo−lysosomal compartments to complement with LgBiT.

(v) The cytosolic transport efficiency was calculated according to the formula in the **Fig. S2**.

The luminescence was measured using microplate reader.

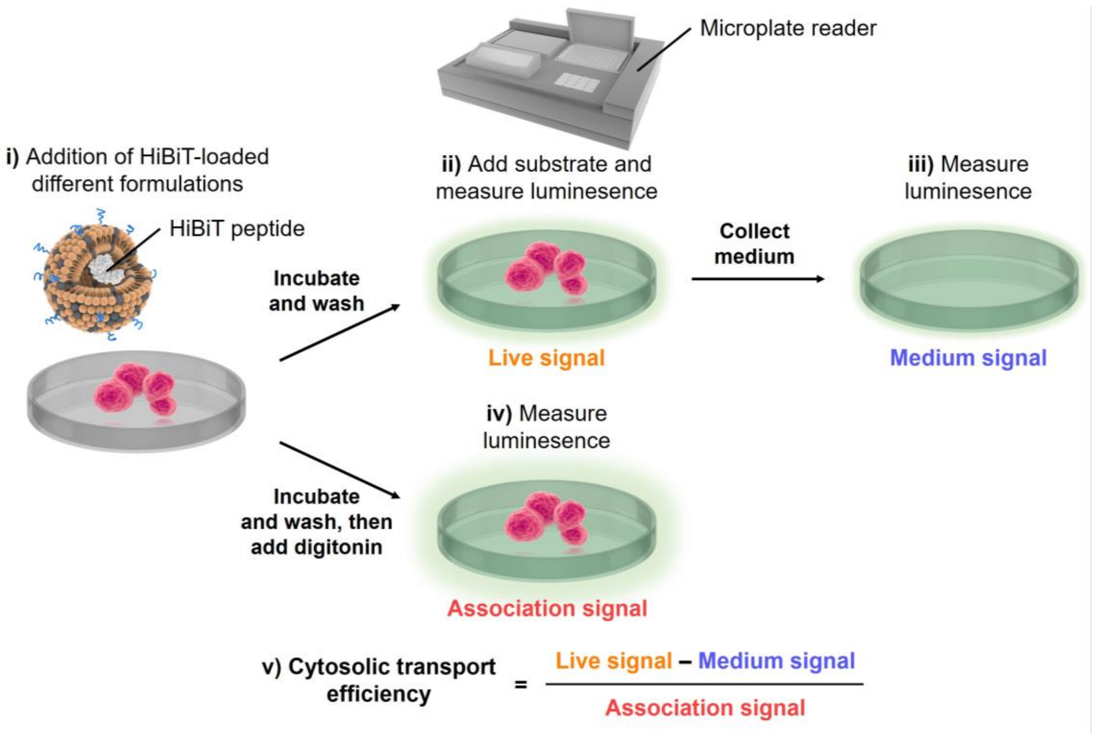

Supplementary Figure 2 | Schematic diagram of the SLEEQ assay.

6. TAzo-LNMs facilitate cross-presentation of tumour antigens

All animal procedures were performed with ethical compliance and approval by the Institutional Animal Care and Use Committee at the Tufts University (Animal Protocol, M2020-51). The female C57BL/6 mice (Charles River) at 6~8-week old were used in this study. Bone marrow-derived dendritic cells (BMDCs) were generated from the bone marrow of C57BL/6 mice.

The BMDCs were incubated with LNM/OVA complexes (OVA, 25 μg mL^{-1}; formulation, 250 μg mL^{-1}) for 6 hours, then received UV/Vis light irradiation for 10 min. After another 18 h incubation, the BMDCs are collected and stained with fluorescent conjugated antibodies for flow cytometry analysis (e.g., PE-anti-mouse CD11C antibody, APC-anti-mouse H2kb-SIINFEKL antibody, FITC-anti-mouse CD80 antibody, and APC-anti-mouse CD86 antibody).

To assess the NLRP inflammasome activation and IL-1β secretion at the cellular level, BMDCs were generated from the bone marrow of C57BL/6 mice. BMDCs were incubated with different LNMs for 6 hours, then received UV/Vis light irradiation for 10 min and replaced with fresh medium. After another 18 h incubation, BMDCs and the culture medium were collected for western blotting immunoassay (WB) and enzyme-linked immunosorbent assay (ELISA).

7. Inhabitation of tumour metastasis to the lung

B16F10-OVA tumour-bearing mice in TAzo-LNM/OVA and TAzo-LNM/OVA + UV/Vis groups were rechallenged with B16F10-OVA cells (1 × 10^6 cells per mouse) via intravenous (IV) injection on day 11. A PBS control group was also treated with B16F10-OVA cells (1 × 10^6 cells per mouse) via IV injection. On day 25, all mice were euthanized and the lungs were collected for photograph.

8. Statistical Analysis

Data were expressed as mean ± SD. All data were analyzed using Graphpad Prism (8.0) software.

9. Supporting results

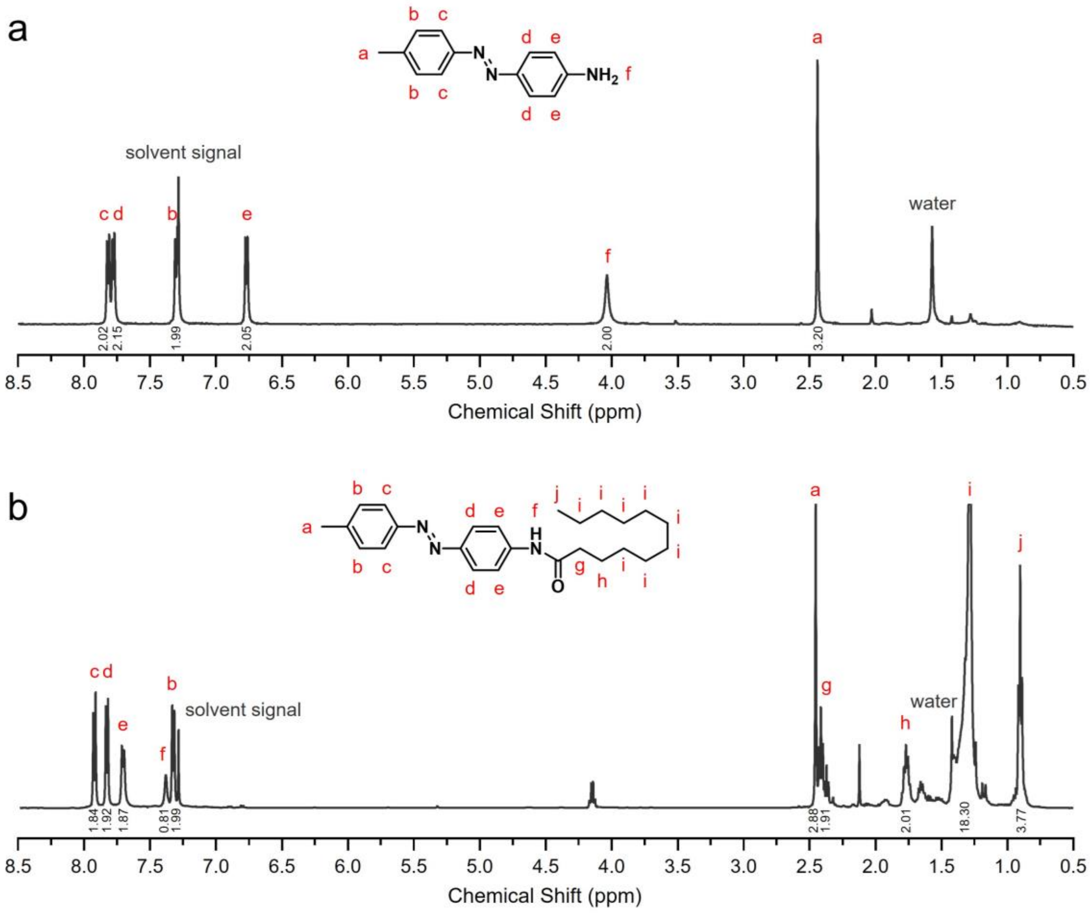

Supplementary Figure 3 | Characterization of Compound 2 and Compound 3. a) ^{1}H NMR spectrum of Compound 2 in $CDCl_3$, 500 MHz, 25 °C. b) ^{1}H NMR spectrum of Compound 3 in $CDCl_3$, 500 MHz, 25 °C.

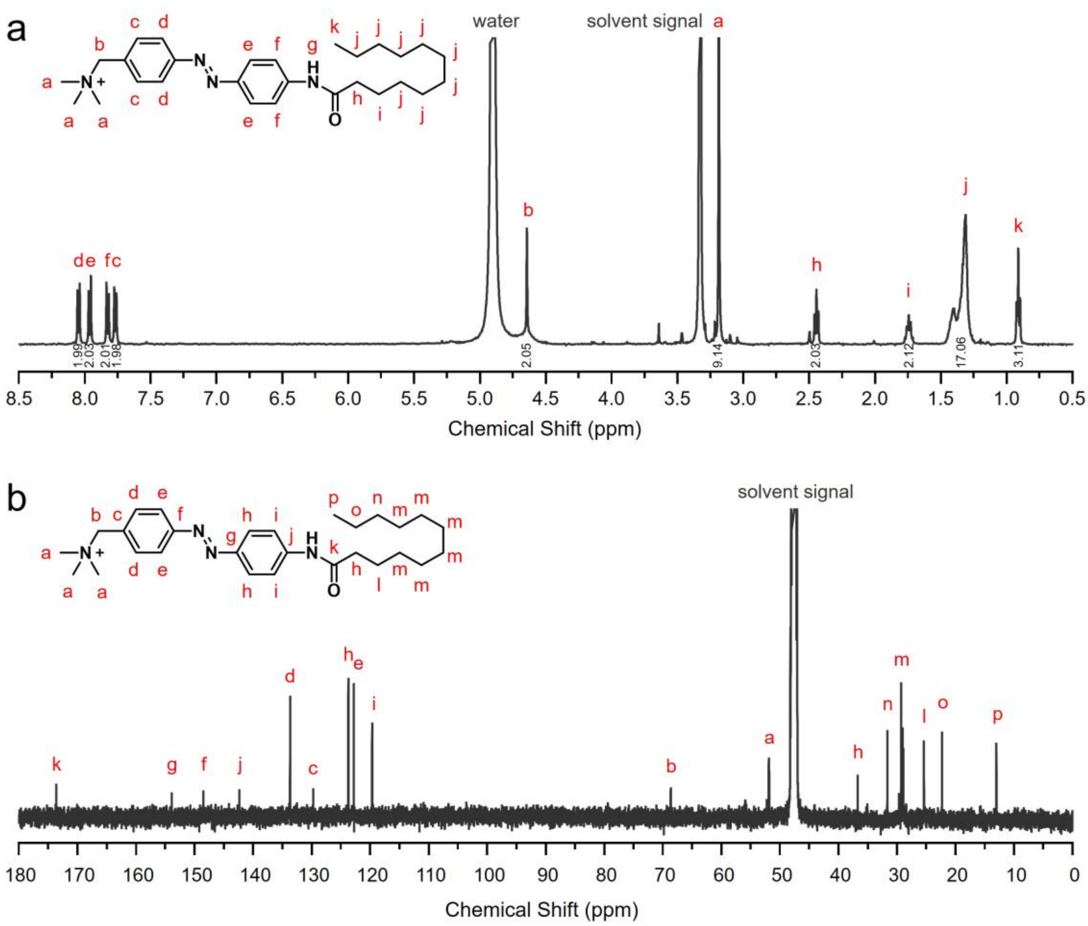

Supplementary Figure 4 | Characterization of SAzo lipidoid. a) ^{1}H NMR spectrum of SAzo lipidoid in CD_3OD, 500 MHz, 25 °C. b) ^{13}C NMR spectrum of SAzo lipidoid in CD_3OD.

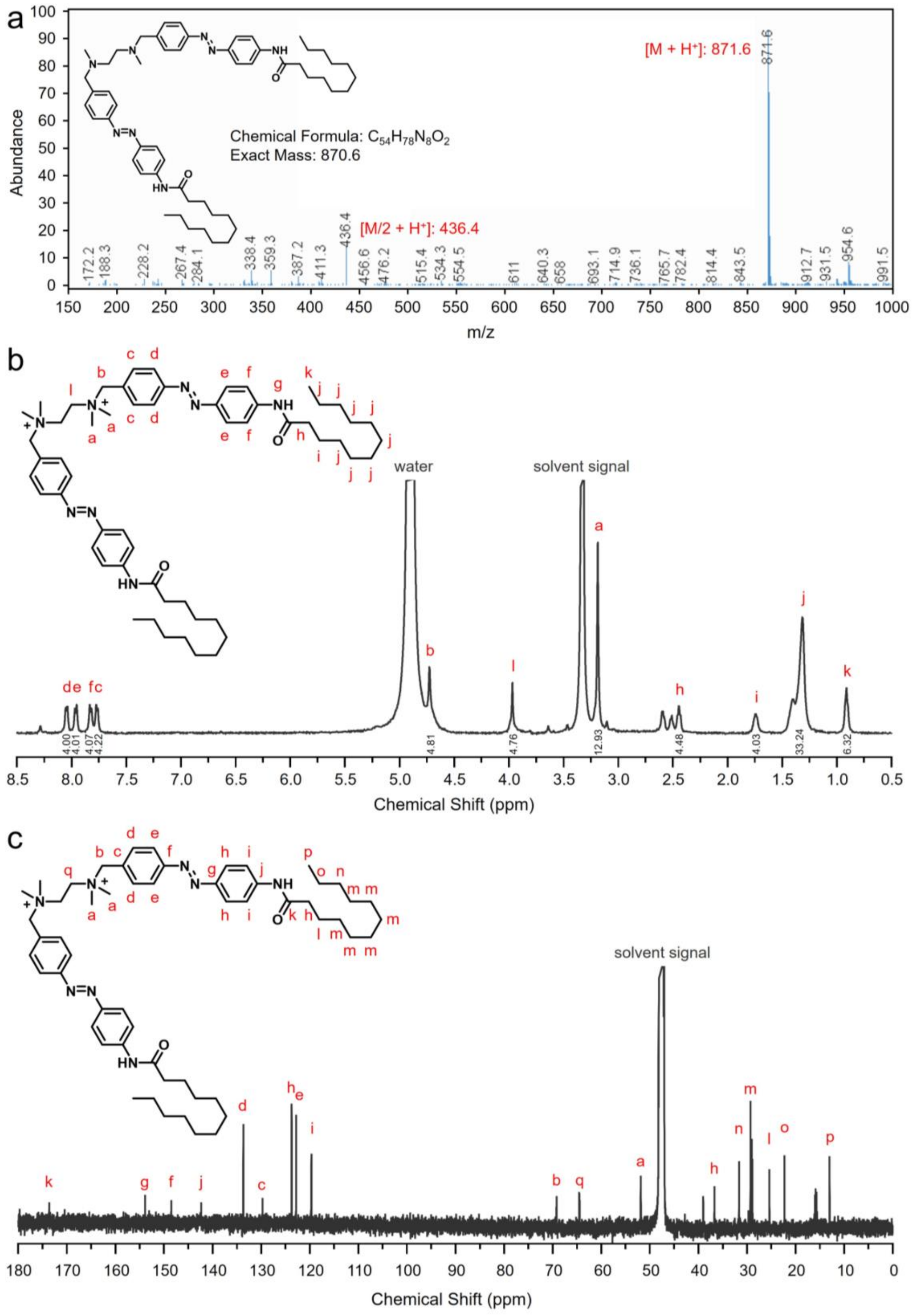

Supplementary Figure 5 | Characterization of TAzo lipidoid. a) HPLC-ESI-MS spectrum of Compound 6. b) ^{1}H NMR spectrum of TAzo lipidoid in CD_3OD, 500 MHz, 25 °C. c) ^{13}C NMR spectrum of TAzo lipidoid in CD_3OD.

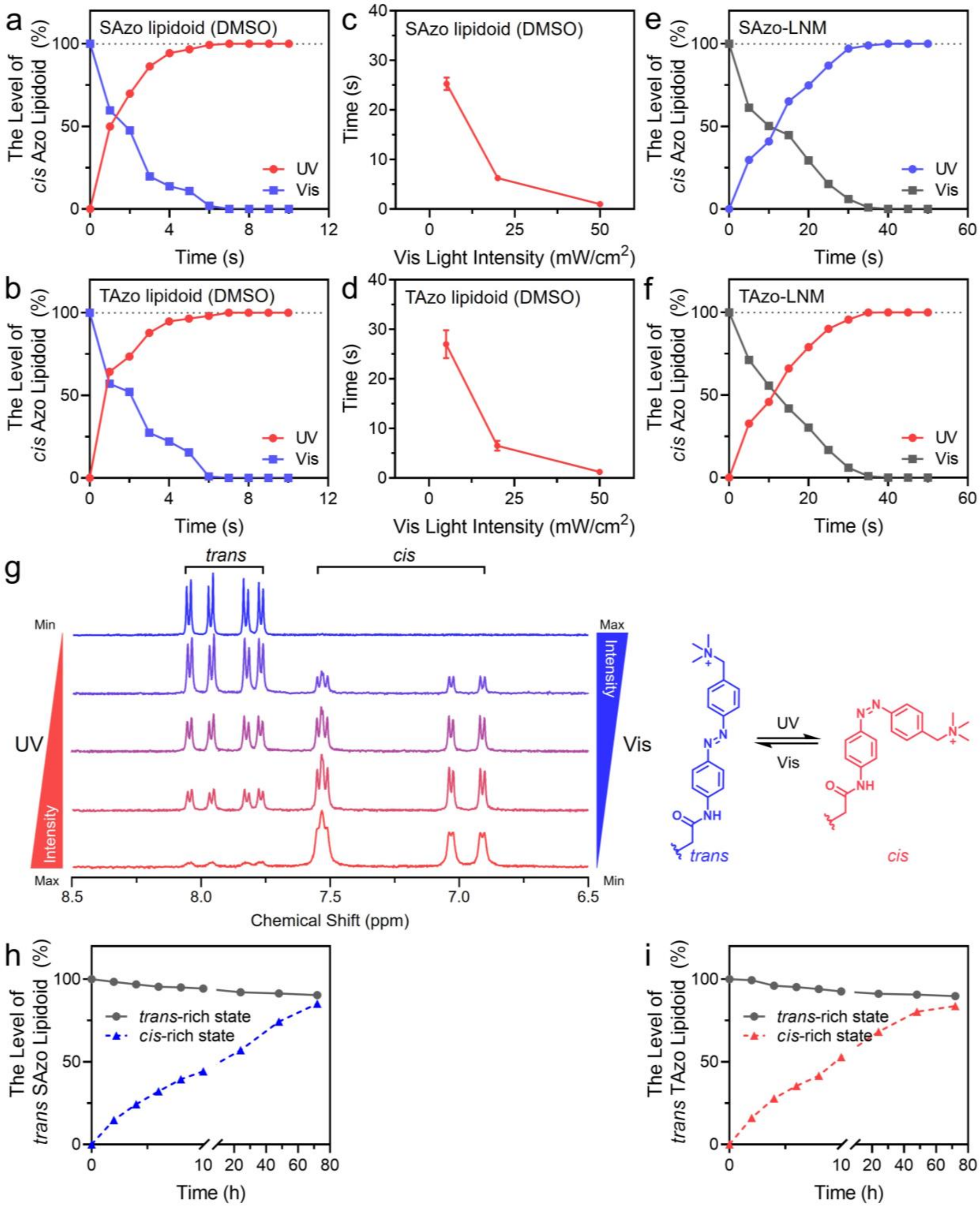

Supplementary Figure 6 | The photoisomerization of Azo-based lipidoids and their LNM formulations. a) and b) The time required to reach the photostationary state in DMSO solution. c) and d) The time required to reach the photostationary state under different Vis light intensity in DMSO solution. e) and f) The time required to reach the photostationary state in PBS at 37 °C. g) ^{1}H NMR spectrum of Azo units in Azo-based lipidoids after simultaneous UV and Vis irradiation. h) and i) Thermal relaxation of different Azo-based lipidoids in dark. Data are presented as mean ± standard deviation (s.d.) from *n* independent experiments (*n* = 3).

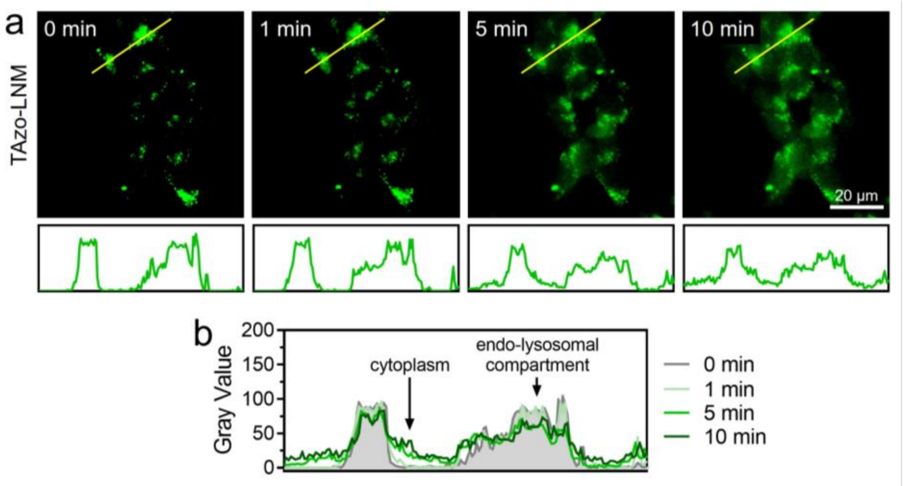

Supplementary Figure 7 | Live-cell imaging experiments show the transport capability of LNMs (TAzo-LNMs). a) Fluorescence images of the cytosolic transport capabilities of NBD-labeled LNMs (TAzo-LNMs) when receiving UV/Vis light irradiation. b) Gray values of green channel (TAzo-LNMs) along with the corresponding yellow solid lines in the fluorescence images.

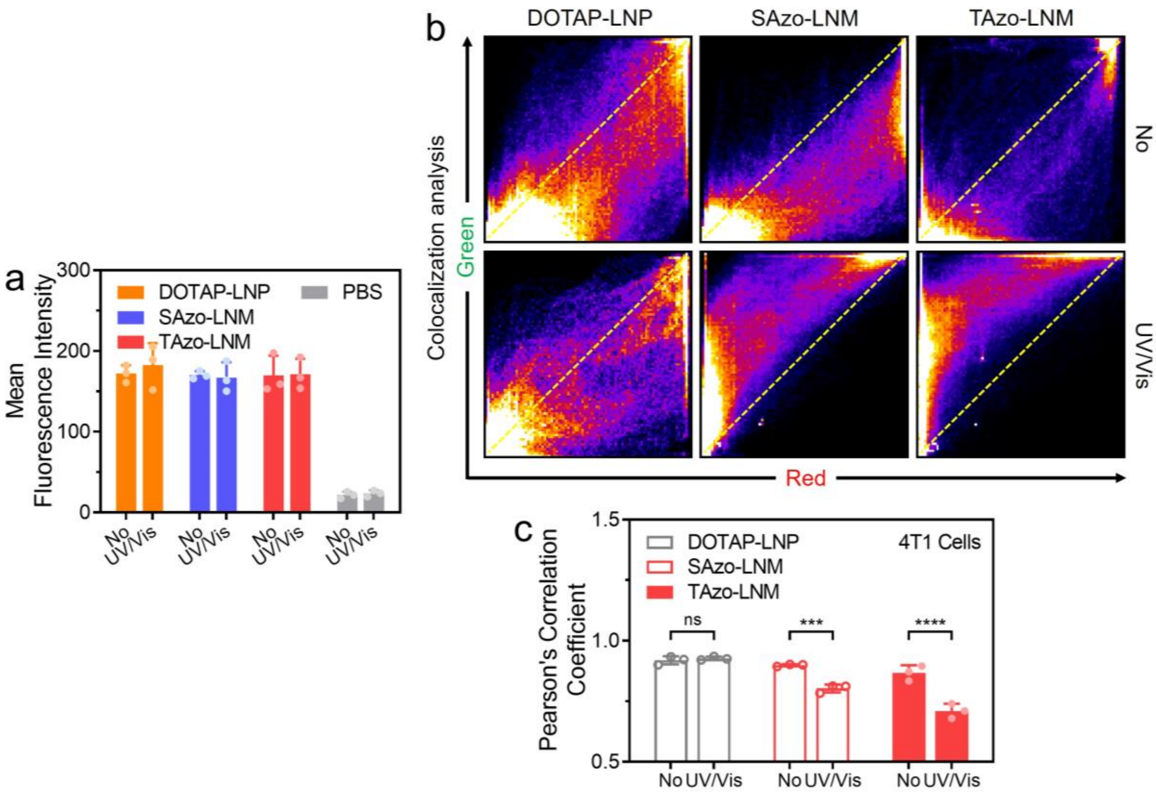

Supplementary Figure 8 | Co-localization analysis of the transport capability of SAzo-LNMs and TAzo-LNMs. a) Flow cytometry analysis of the internalization of NBD-labeled formulations in HeLa cells. b) and c) Quantitative analysis of co-localization of NBD-labeled formulations with endo−lysosomal compartments labelled with LysoTracker Red. The coefficients are close to 1 if they are highly colocalized. Data are presented as mean ± standard deviation (s.d.) from *n* independent experiments ($n = 3$). Statistical significance was analyzed by two-way ANOVA with Sidak's multiple comparisons test. ($^{***}p < 0.001$, $^{****}p < 0.0001$).

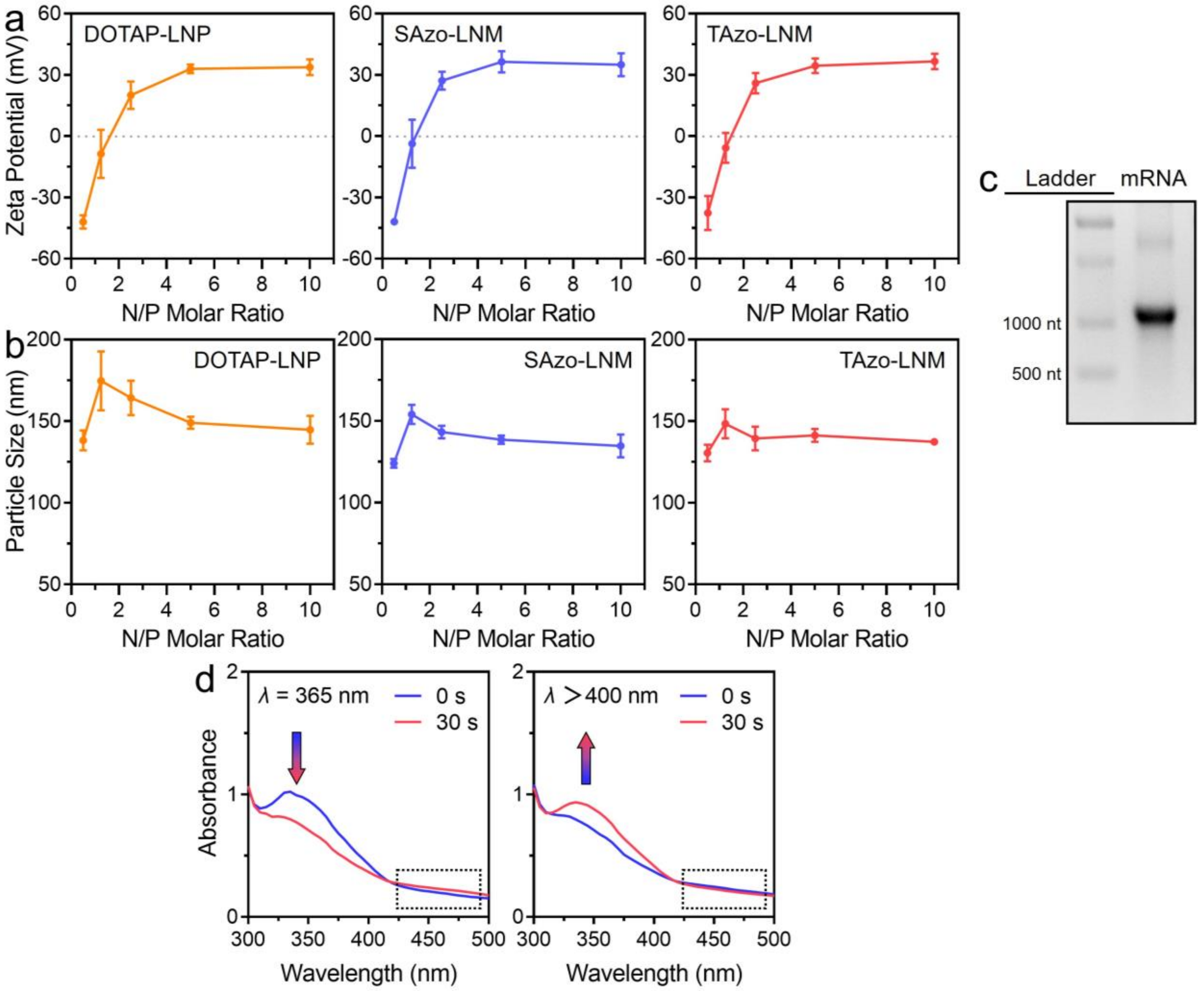

Supplementary Figure 9 | Characterization of GFP mRNA-loaded SAzo-LNMs and TAzo-LNMs. a) Zeta potentials and b) particle size of different GFP mRNA-loaded formulations at five different N/P ratios (from 0.5 to 10). c) Agarose gel electrophoresis of GFP mRNA (2% TAE gel electrophoresis, 750 ng mRNA loaded for assay). d) UV−Vis absorption spectrum of GFP mRNA-loaded TAzo-LNMs after different treatments. The samples are irradiated with UV and Vis light for 30 s in PBS at 37 °C, respectively. Data are presented as mean ± standard deviation (s.d.) from *n* independent experiments (*n* = 3).

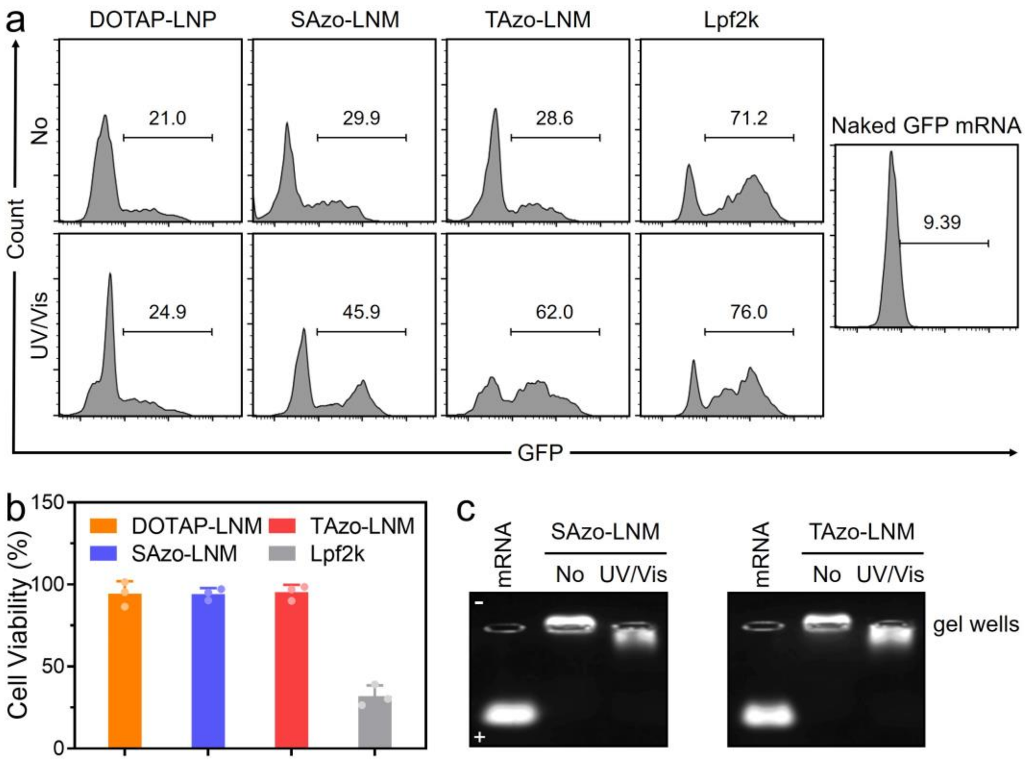

Supplementary Figure 10 | LNMs transport GFP mRNA from endo−lysosomal compartments to cytoplasm. a) GFP mRNA transfection efficacy of DOTAP-LNP, SAzo-LNM, TAzo-LNM, Lpf2k, and naked mRNA tested on HeLa cells by quantifying GFP-positive cells. b) Cell viability of HeLa cells after 24 h incubation with of GFP mRNA-loaded DOTAP-LNP, SAzo-LNM, TAzo-LNM, and Lpf2k (mRNA, 1 µg mL^{-1}; formulation, 50 µg mL^{-1}), respectively. c) Agarose gel electrophoresis of GFP mRNA-loaded different LNMs after UV/Vis irradiation. Data are presented as mean ± standard deviation (s.d.) from *n* independent experiments (*n* = 3).

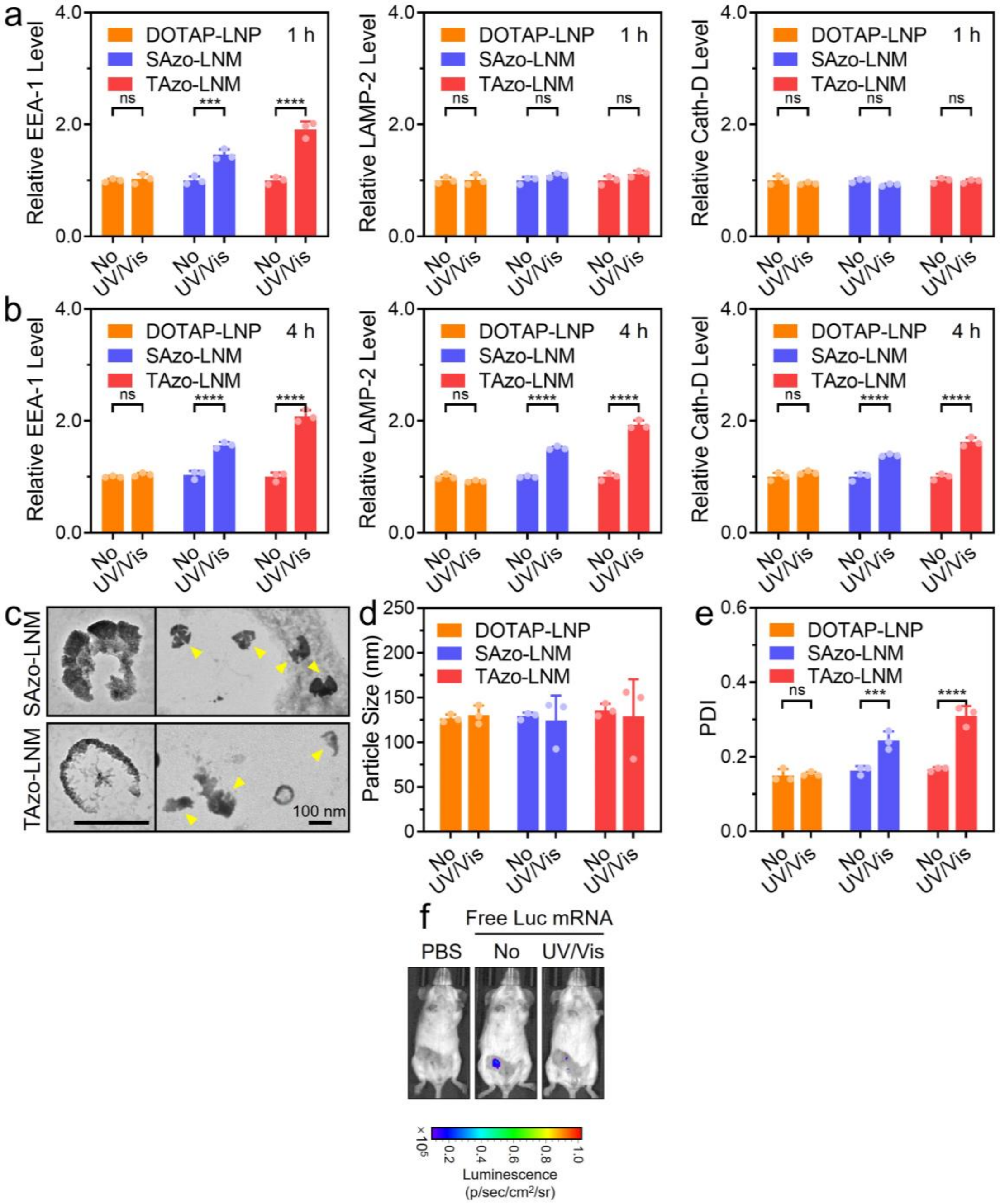

Supplementary Figure 11 | LNMs destabilize the endo−lysosomal membrane and induce membrane disruption. a) and b) Detection of EEA-1, LAMP-2, and Cath-D in the cytoplasm. c) TEM images of SAzo-LNMs and TAzo-LNMs after UV/Vis irradiation. Scale bar, 100 μm. d) The changes of particle size of DOTAP-LNP, SAzo-LNM, and TAzo-LNM after UV/Vis irradiation. e) The changes of PDI values of DOTAP-LNP, SAzo-LNM, and TAzo-LNM after UV/Vis irradiation. f) Representative whole-body bioluminescence images of mice after SC injection of Luc mRNA (0.25 mg kg^{-1}, 5 μg per mouse) measured by the IVIS imaging system. Images were taken at 5 h post-injection. Data are presented as mean ± standard deviation (s.d.) from *n* independent experiments (*n* = 3). Statistical significance was analyzed by two-way ANOVA with Sidak's multiple comparisons test. ($^{*}p < 0.05$, $^{***}p < 0.001$, $^{****}p < 0.0001$).

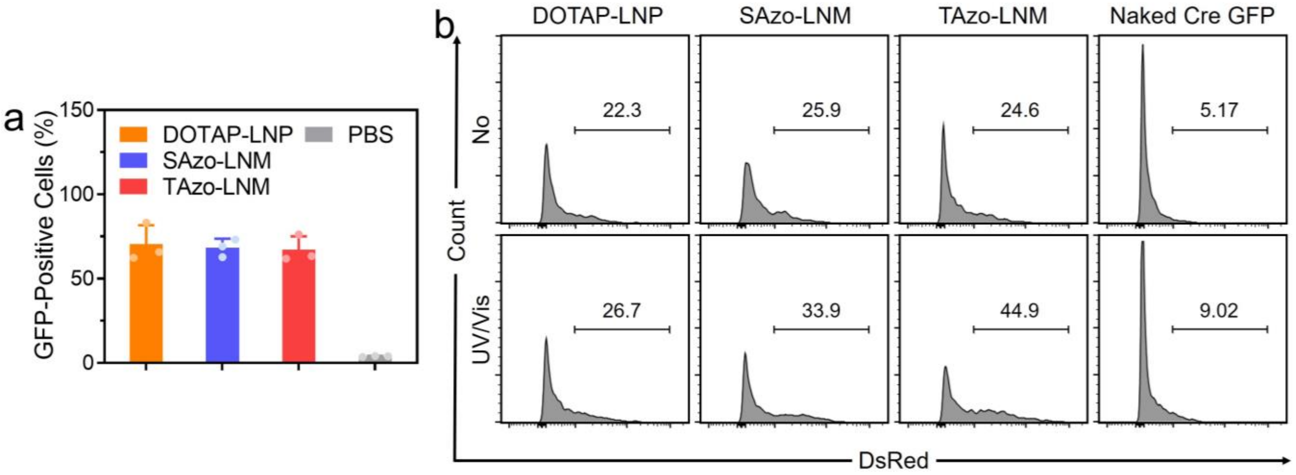

Supplementary Figure 12 | LNMs transport Cre proteins from endo−lysosomal compartments to cytoplasm. a) Flow cytometry analysis of the internalization of LNM/(−30)GFP-Cre complexes (1.5 μg mL^{-1} protein) in HeLa cells after 30 min incubation. b) DsRed expression efficacy of DOTAP-LNP/(−30)GFP-Cre, SAzo-LNM/(−30)GFP-Cre, TAzo-LNM/(−30)GFP-Cre, and naked (−30)GFP-Cre (1.5 μg mL^{-1} protein) tested on HeLa-DsRed cells after 24 h incubation, respectively. Data are presented as mean ± standard deviation (s.d.) from *n* independent experiments (*n* = 3).

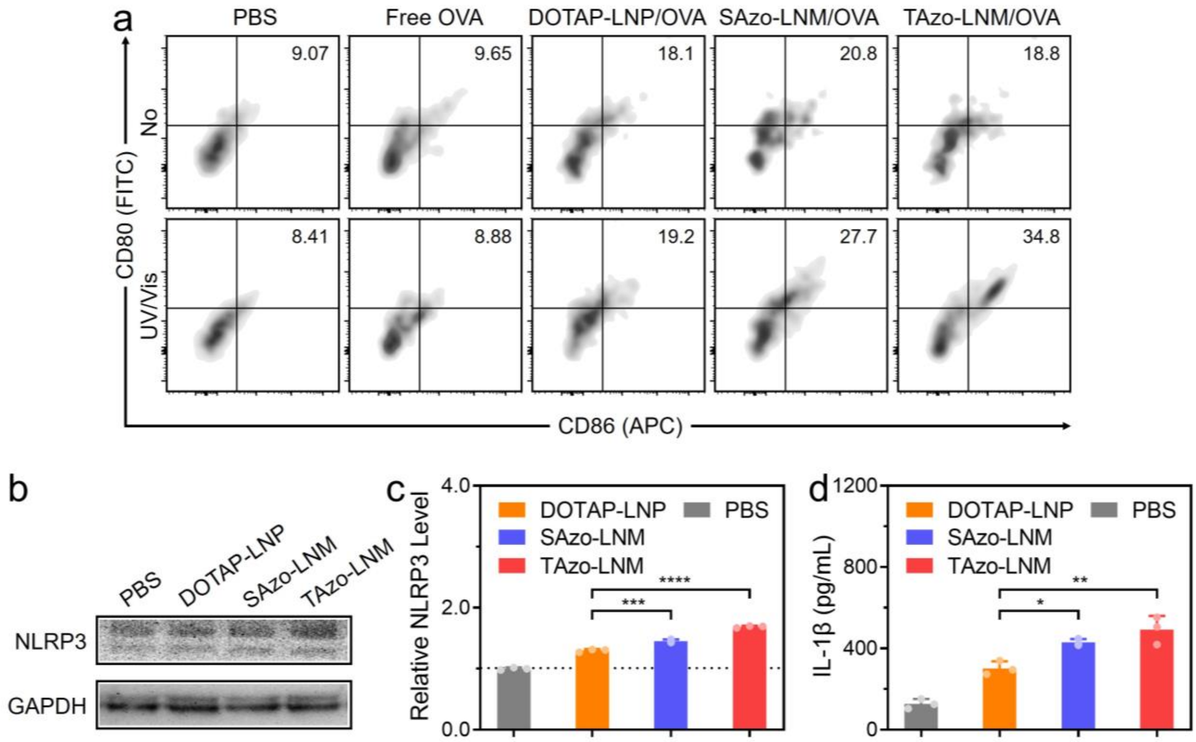

Supplementary Figure 13 | Analysis of DC maturation. a) Representative flow cytometry analysis of the population of CD80^{+}CD86^{+} BMDCs after different treatments. b) and c) Detection of NLRP3 expression in BMDCs after different treatments using western blotting analysis. d) IL-1β secretion in BMDCs. Data are presented as mean ± standard deviation (s.d.) from *n* independent experiments ($n = 3$). Statistical significance was analyzed by one-way ANOVA with Tukey's multiple comparisons test. ($^{*}p < 0.05$, $^{**}p < 0.01$, $^{***}p < 0.001$, $^{****}p < 0.0001$).

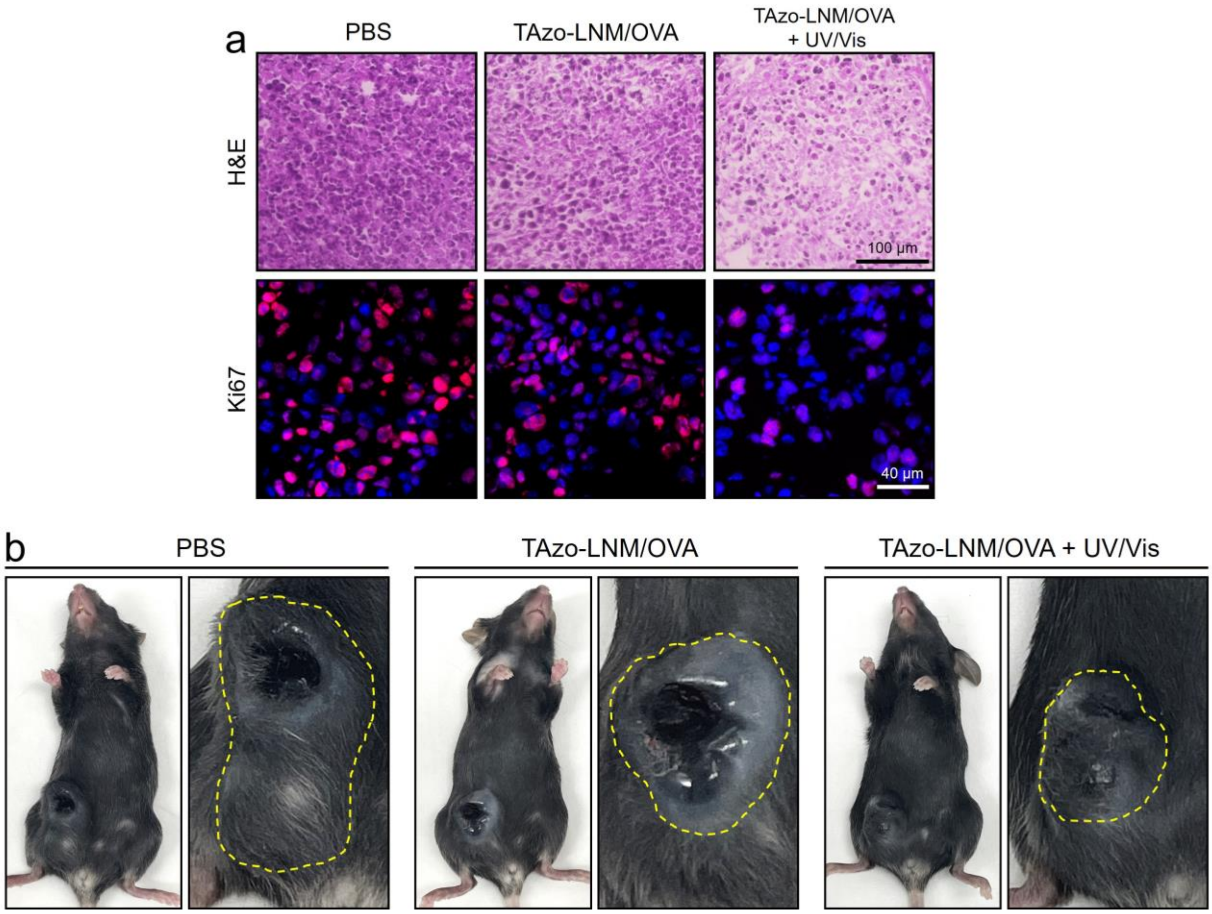

Supplementary Figure 14 | Antitumour effect in a mouse model of melanoma. a) hematoxylin and eosin (H&E) staining and cell proliferation antigen Ki-67 staining. b) The photographs of B16F10-OVA tumours.

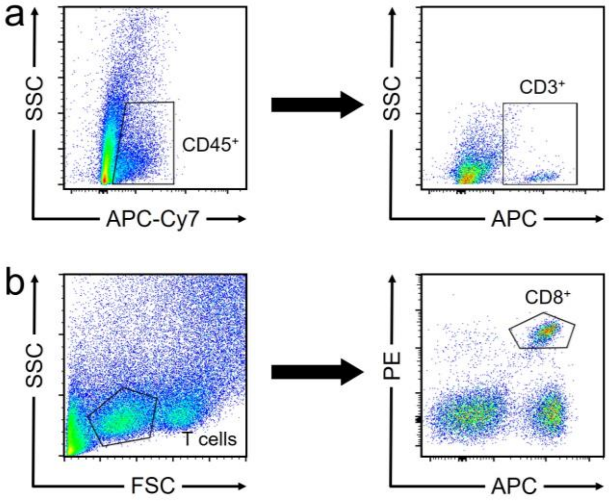

Supplementary Figure 15 | Gating strategy for identification of lymphocytes. a) Gating strategy for identification of $CD3^+$ T cells within the tumour tissues. b) Gating strategy for identification of $CD8^+$ T cells in the spleens.

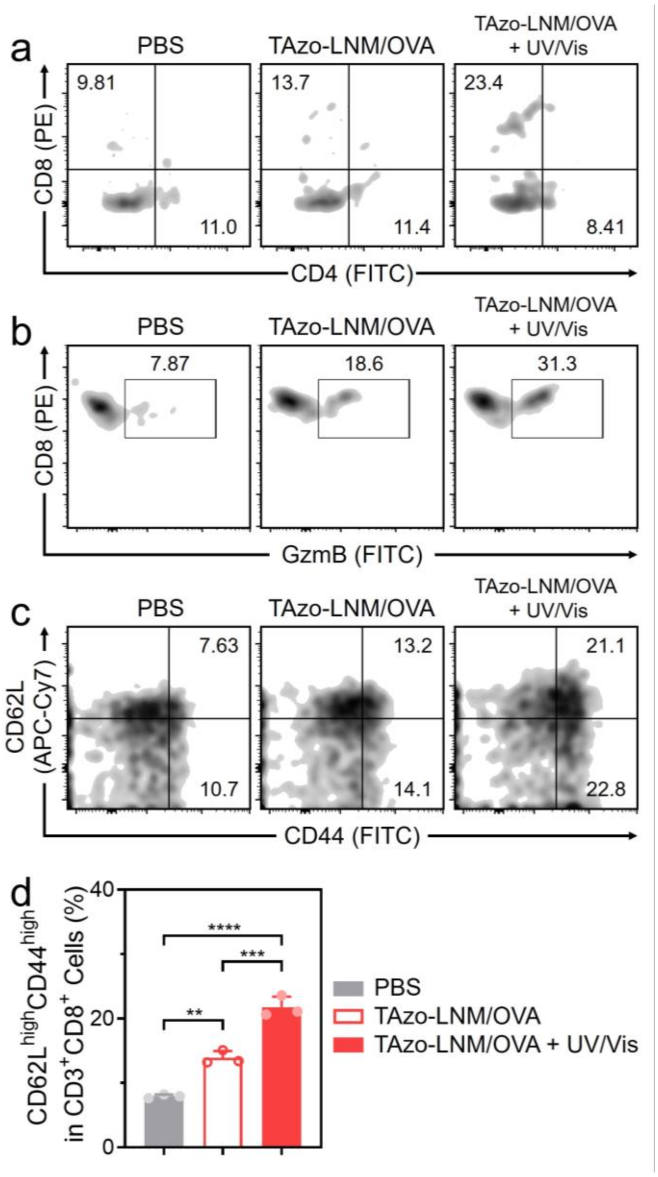

Supplementary Figure 16 | Antitumour immunity *in vivo*. a) Representative flow cytometry analysis of the population of $CD8^+$ T cells (gated on $CD45^+CD3^+$ cells) in tumour tissues after different treatments. b) Representative flow cytometry analysis of the expression of $GzmB^{high}$ in $CD8^+$ T cells (gated on $CD45^+CD3^+$ cells) in tumour tissues. c) Representative flow cytometry analysis of the $CD62L^{low}CD44^{high}$ and $CD62L^{high}CD44^{high}$ T cells (gated on $CD3^+CD8^+$ cells) in the spleens. d) Flow cytometry analysis of the $CD62L^{high}CD44^{high}$ T cells (gated on $CD3^+CD8^+$ cells) in the spleens. Data are presented as mean ± standard deviation (s.d.) from *n* independent experiments ($n = 3$). Statistical significance was analyzed by one-way ANOVA with Tukey's multiple comparisons test. ($^{**}p < 0.01$, $^{***}p < 0.001$, $^{****}p < 0.0001$).

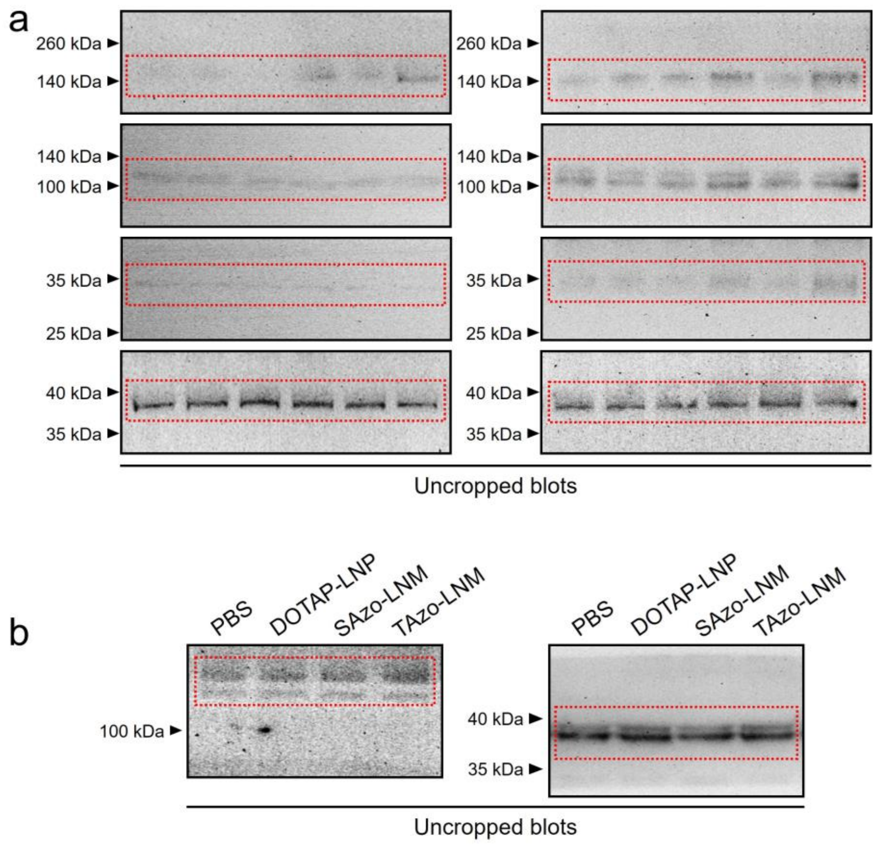

Supplementary Figure 17 | Western blots assay. a) Western blots for Figure 4c and 4d. b) Western blots for Figure S13b.

10. Supplementary References

1. Zhao, Y. et al. Lipidoid Artificial Compartments for Bidirectional Regulation of Enzyme Activity through Nanomechanical Action. *J. Am. Chem. Soc.* **145**, 551-559 (2023).
2. Teo, S.L. et al. Unravelling cytosolic delivery of cell penetrating peptides with a quantitative endosomal escape assay. *Nat. Commun.* **12**, 3721 (2021).
